# 5-Hydroxymethyltubercidin exhibits potent antiviral activity against flaviviruses and coronaviruses, including SARS-CoV-2

**DOI:** 10.1016/j.isci.2021.103120

**Published:** 2021-09-11

**Authors:** Kentaro Uemura, Haruaki Nobori, Akihiko Sato, Takao Sanaki, Shinsuke Toba, Michihito Sasaki, Akiho Murai, Noriko Saito-Tarashima, Noriaki Minakawa, Yasuko Orba, Hiroaki Kariwa, William W. Hall, Hirofumi Sawa, Akira Matsuda, Katsumi Maenaka

**Affiliations:** 1 Drug Discovery and Disease Research Laboratory, Shionogi & Co., Ltd., Osaka, Japan; 2 Division of Molecular Pathobiology, International Institute for Zoonosis Control, Hokkaido University, Sapporo, Japan; 3 Laboratory of Biomolecular Science, Faculty of Pharmaceutical Sciences, Hokkaido University, Sapporo, Japan; 4 Graduate School of Pharmaceutical Science, Tokushima University, Tokushima, Japan; 5 International Collaboration Unit, International Institute for Zoonosis Control, Hokkaido University, Sapporo, Japan; 6 Laboratory of Public Health, Graduate School of Veterinary Medicine, Hokkaido University, Sapporo, Japan; 7 National Virus Reference Laboratory, School of Medicine, University College of Dublin, Ireland; 8 Global Virus Network, Baltimore, MD, USA; 9 One Health Research Center, Hokkaido University, Sapporo, Japan; 10 Center for Research and Education on Drug Discovery, Faculty of Pharmaceutical Sciences, Hokkaido University, Sapporo, Japan; 11 Global Station for Biosurfaces and Drug Discovery, Hokkaido University, Sapporo, Japan

**Keywords:** Drugs, Microbiology, Viral microbiology

## Abstract

Newly emerging or re-emerging viral infections continue to cause significant morbidity and mortality every year worldwide, resulting in serious effects on both health and the global economy. Despite significant drug discovery research against dengue viruses (DENVs) and severe acute respiratory syndrome coronavirus-2 (SARS-CoV-2), no fully effective and specific drugs directed against these viruses have been discovered. Here, we examined the anti-DENV activity of tubercidin derivatives from a compound library from Hokkaido University and demonstrated that 5-hydroxymethyltubercidin (HMTU, HUP1108) possessed both potent anti-flavivirus and anti-coronavirus activities at submicromolar levels without significant cytotoxicity. Furthermore, HMTU inhibited viral RNA replication and specifically inhibited replication at the late stages of the SARS-CoV-2 infection process. Finally, we demonstrated that HMTU 5′-triphosphate inhibited RNA extension catalyzed by the viral RNA-dependent RNA polymerase. Our findings suggest that HMTU has the potential of serving as a lead compound for the development of a broad spectrum of antiviral agents, including SARS-CoV-2.

## Highlights

• We identified tubercidin derivatives as broad-spectrum antiviral compounds
• HMTU exhibited potent antiviral activity against flaviviruses and coronaviruses
• HMTU specifically inhibited replication at a late stage of the viral infection process
• HMTU 5′-triphosphate inhibited RNA extension catalyzed by viral RdRp

## Graphical abstract

**Figure:**
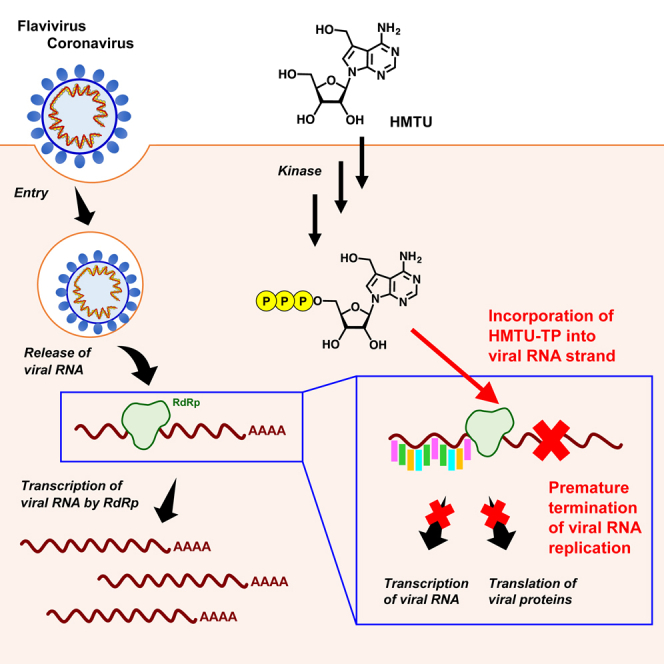

## Introduction

Newly emerging and re-emerging viral infectious diseases have been found to have a significant negative impact on global human health and global economies. Despite significant progress in medical treatment and the pharmacological sciences, these viral infections continue to cause millions of cases of disease and deaths worldwide annually.

Since the first reported case in December 2019, severe acute respiratory syndrome coronavirus-2 (SARS-CoV-2) has rapidly spread from person to person, and SARS-CoV-2 infection is now pandemic. The severe pneumonia-like disease caused by SARS-CoV-2, designated as coronavirus disease 2019 (COVID-19), has been linked to more than 191 million confirmed cases and 4.1 million deaths in 192 countries/regions [https://coronavirus.jhu.edu/map.html]. SARS-CoV-2 is a member of the *Betacoronavirus* genus of the family *Coronaviridae*, which consists of several types of human coronaviruses (HCoVs), including HCoV-229E, HCoV-NL63, HCoV-OC43, HCoV-HKU1, SARS-CoV, and Middle East respiratory syndrome coronavirus (MERS-CoV).

Coronaviruses are enveloped, approximately 30 kb positive-sense, single-stranded RNA viruses that can infect a wide range of animal species, in addition to humans (Cui et al., 2019). To date, many compound-repurposing studies against SARS-CoV-2 have been performed (Ohashi et al., 2021; Riva et al., 2020; Wang et al., 2020), and several candidate compounds, including remdesivir, have been evaluated in clinical trials for the treatment of patients with COVID-19 (Grein et al., 2020; Li and De Clercq, 2020). However, there are as yet no fully effective specific drugs directed against SARS-CoV-2, and as such, effective therapeutic agents and vaccines against this virus are urgently needed.

Dengue virus (DENV) causes widespread endemic disease known as dengue fever and dengue hemorrhagic fever, resulting in an estimated 390 million total infections, 100 million clinical cases, and 500,000 severe cases per year worldwide (Pierson and Diamond, 2020). DENV is made up of four serotypes, DENV1-4, which are members of the *Flavivirus* genus of the family *Flaviviridae*. This family includes several other significant human pathogens, such as Zika virus (ZIKV), yellow fever virus (YFV), Japanese encephalitis virus (JEV), and West Nile virus (WNV) (Pierson and Diamond, 2020). Flaviviruses are also enveloped and have an approximate 11 kb positive-sense, single-stranded RNA that can be transmitted between arthropods and humans (Simmonds et al., 2017; Vasilakis and Weaver, 2017). Dengue is defined as a neglected tropical disease (NTD) by the World Health Organization (Mackey and Liang, 2012), and there are yet no fully effective drugs or vaccines for treatment or prevention. Furthermore, there are as yet currently no specific agents that are fully effective against the other flaviviruses. As such, effective therapeutic agents and vaccines against all flaviviruses are also urgently needed.

Nucleos(t)ides and their analogs are widely reported to exhibit antiviral activities; thus, we have now examined the anti-DENV activity of an original nucleoside analog library from the Center for Research and Education on Drug Discovery of Hokkaido University in Japan. Tubercidin (7-deazaadenosine) showed anti-DENV activity. Tubercidin was identified as an antibiotic obtained from the bacteria *Streptomyces tubercidicus* (Suzuki and Marumo, 1960), and this compound with its derivatives has been reported to exhibit antiviral activity against several viruses including vesicular stomatitis virus (VSV), coxsackie virus, polio virus, rhinovirus, and ZIKV (Bergstrom et al., 1984; De Clercq et al., 1986; Eyer et al., 2016). In addition, recently, the antiviral activities of 5-substituted tubercidin derivatives against emerging RNA viruses have been reported (Milisavljevic et al., 2021). In this study, we further examined the antiviral activity of tubercidin derivatives and have demonstrated that 5-hydroxymethyltubercidin (HMTU, HUP1108) possessed potent anti-flavivirus and anti-coronavirus activities without obvious cytotoxicity. Furthermore, we examined the specific antiviral activity of HMTU against SARS-CoV-2 and SARS-CoV. We also performed real-time quantitative reverse transcription PCR (qRT-PCR) analysis, a 50% tissue culture infective dose (TCID_50_) assay, immunofluorescence assays (IFAs), and the time-of-addition assay to better understand how HMTU affects the replication cycles of these viruses. Finally, we performed RNA extension assays using the viral RNA-dependent RNA polymerase (RdRp) which revealed the incorporation of HMTU 5′-triphosphate (HMTU-TP) into viral RNA, resulting in chain termination of the viral RNA.

## Results

### Antiviral activities of tubercidin derivatives on flaviviruses and human coronaviruses

**Figure 1:**
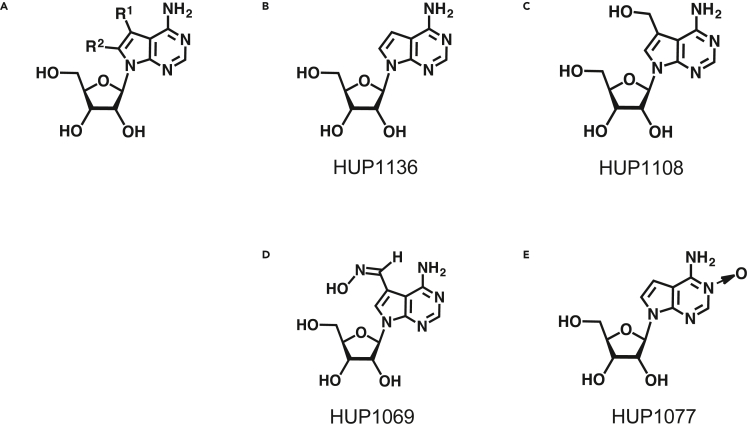
Chemical structures of tubercidin and tubercidin derivatives (A) General structure of tubercidin. (B) Chemical structure of tubercidin (HUP1136). (C) Chemical structure of 5-hydroxymethyltubercidin (HUP1108, HMTU). (D) Chemical structure of 5-formyltuberucidin oxime (HUP1069). (E) Chemical structure of 3-tubercidin N-oxide (HUP1077).

In this study, we initially screened 753 nucleoside analogs in a compound library from Hokkaido University. The half maximal effective concentration (EC_50_) and half maximal cytotoxic concentration (CC_50_) values of these compounds were initially determined using DENV2 in BHK-21 cells. From the results of the 3-[4,5-dimethyl-2-thiazolyl]-2,5-diphenyl-2H-tetrazolium bromide (MTT) assay (Nobori et al., 2018), we selected four tubercidin derivatives with potent anti-DENV2 activity for further studies (Figure 1A; Table 1): HUP1136 (tubercidin, EC_50_ < 0.125 μM, Figure 1B), HUP1108 (HMTU, EC_50_ = 0.24 μM, Figure 1C), HUP1069 (5-formyltuberucidin oxime, EC_50_ < 0.125 μM, Figure 1D), and HUP1077 (3-tubercidin N-oxide, EC_50_ = 0.95 μM, Figure 1E). Furthermore, cytotoxicity assays were performed at the same time to determine the toxicity of these compounds. Although HUP1136 and HUP1069 showed high cytotoxicity, HUP1108 and HUP1077 did not exhibit cytotoxicity against BHK-21 cells at concentrations up to 20 μM (Table 1). Subsequently, we evaluated the antiviral activities of HUP1108 against other flaviviruses (ZIKV, YFV, JEV, and WNV), and HUP1108 was shown to inhibit all of these flaviviruses, as shown in Table 2. The results suggested that tubercidin and tubercidin derivatives are potentially broad-spectrum antiviral agents, and these findings are consistent with those described in previous reports (Eyer et al., 2016; Milisavljevic et al., 2021; Olsen et al., 2004).

**Table 1:** EC_50_ and CC_50_ values of tubercidin derivatives in DENV2-infected BHK-21 cells

Compound ID	R^1^	R^2^		DENV2	CC_50_ (μM)
				EC_50_ (μM)	
HUP1136	H	H		<0.125	0.34
HUP1108	CH_2_OH	H		0.24	>20
HUP1069	CH=NOH	H		<0.125	0.18
HUP1077	H	H	N^3^-O	0.95	>20
Ribavirin				28	>400

**Table 2:** EC_50_ Values of HUP1108 (HMTU) against flaviviruses

Virus	Cell line	Time	Assay	HUP1108 EC_50_ (μM)	Ribavirin EC_50_ (μM)	Favipiravir EC_50_ (μM)
DENV1	BHK-21	5 days	Resazurin	0.35	62	17
DENV2	BHK-21	4 days	Resazurin	0.24	19	4.4
DENV3	BHK-21	5 days	Resazurin	0.42	54	13
DENV4	BHK-21	5 days	Resazurin	0.52	86	5.2
ZIKV	BHK-21	3 days	MTT	1.3	31	40
YFV	BHK-21	3 days	MTT	0.27	34	57
JEV	BHK-21	3 days	MTT	0.50	33	80
WNV	BHK-21	3 days	MTT	0.95	52	>100

In view of the circumstances underlying the COVID-19 pandemic, we rescreened nine tubercidin derivatives, including the above four named compounds against human coronaviruses using the resazurin reduction assay (Sanaki et al., 2019). We also examined whether these tubercidin derivatives inhibited SARS-CoV-2 replication using real-time qRT-PCR analysis. In the resazurin reduction assay using MRC5 cells, HUP1136, HUP1108, HUP1069, and HUP1077 exhibited strong antiviral activities against both HCoV-OC43 and HCoV-229E, whereas no or only low anti-HCoV activities of other tubercidin derivatives were observed (Table 3). In the qRT-PCR analysis using Caco-2 cells, HUP1136 (EC_90_ = 0.087 μM), HUP1108 (EC_90_ = 0.57 μM), HUP1069 (EC_90_ = 0.20 μM), and HUP1077 (EC_90_ = 3.5 μM) inhibited SARS-CoV-2 infection (Table 3). As noted above, HUP1136 and HUP1069 showed high cytotoxicity in MRC5 cells. Furthermore, the antiviral activities of HUP1077 were lower than those of the other three compounds. Thus, we selected HUP1108 (HMTU) for further analysis of activity against human coronaviruses.

**Table 3:** EC_50_ and CC_50_ values of tubercidin derivatives in HCoV-infected MRC5 cells and EC_90_ values against SARS-CoV-2

Compound ID	R^1^	R^2^		HCoV-OC43	HCoV-229E	CC_50_ (μM)^b^	SARS-CoV-2
				EC_50_ (μM)^a^	EC_50_ (μM)^a^		EC_90_ (μM)^c^
HUP1136	H	H		0.11	<0.078	0.66	0.087
HUP1108	CH_2_OH	H		0.35	0.43	>50	0.57
HUP1069	CH=NOH	H		0.055	<0.078	0.74	0.20
HUP1077	H	H	N^3^-O	4.3	4.0	24	3.5
HUP1078	NO_2_	H		>10	2.0	0.33	0.10
HUP1079	CH_3_	H		>10	>10	>50	>10
HUP1109	CN	H		>10	2.3	0.59	0.20
HUP1111	H	CN		>10	>10	>50	ND^d^
HUP1070	1-Morpholinomethyl	Br		>10	>10	>50	ND^d^

a Resazurin reduction assay using MRC5 cells.

b Cytotoxicity against MRC5 cells.

c qRT-PCR analysis using Caco-2 cells.

d ND indicates not determined.

### HMTU inhibits HCoV replication

**Figure 2:**
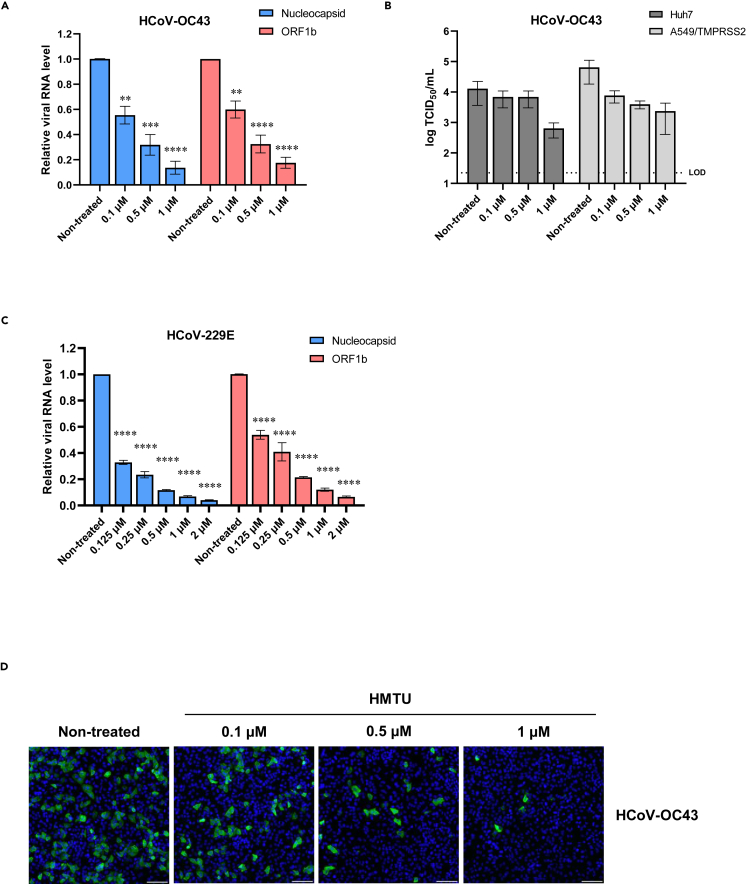
Antiviral activities of HMTU against HCoVs *in vitro* (A and C) Viral RNA expression was quantified by qRT-PCR analysis. Huh7 cells were infected with HCoV-OC43 (A) or HCoV-229E (C) at a multiplicity of infection (MOI) of 0.1 or 0.05, respectively, containing a serially diluted compound. At 48 hpi, total RNA was collected, and the relative expression of the nucleocapsid and ORF1b genes was evaluated by qRT-PCR with β-actin mRNA used as a reference control. Data represent mean values of at least three independent experiments, and error bars indicate standard error of the mean (SEM). Statistically significant differences were determined using a one-way ANOVA followed by Dunnett's multiple comparisons test; ∗∗p < 0.005, ∗∗∗p < 0.0005, and ∗∗∗∗p < 0.0001. (B) Progeny virus titers were quantified by the TCID_50_ assay. Huh7 and A549/TMPRSS2 cells were infected with HCoV-OC43 at an MOI of 0.1 containing a serially diluted compound. At 48 hpi, supernatants were collected, and dilutions were used to inoculate MRC5 cells. Three days after inoculation, viral titers were determined by calculation of TCID_50_/mL. Data represent mean values of at least three independent experiments, and error bars indicate SEM. The dotted line indicates the limit of detection (LOD). (D) Viral nucleocapsid protein expression in the infected cells. A549/TMPRSS2 cells were infected with HCoV-OC43 at an MOI of 1 containing a serially diluted compound. At 48 hpi, cells were stained with an anti-coronavirus nucleocapsid protein antibody (green) and counterstained with Hoechst 33342 nuclear dye (blue). Scale bars indicate 200 μm.

To evaluate the effects of HMTU on HCoV infections, we performed a resazurin reduction assay using MRC5 cells and compared the anti-coronaviral effect of HMTU with that of a Food and Drug Administration (FDA)-approved drug, remdesivir. The results showed that the EC_50_ of HMTU against HCoV-OC43 and HCoV-229E was 0.378 ± 0.023 and 0.528 ± 0.029 μM, respectively (Table 4). Importantly, the CC_50_ of HMTU obtained from the MRC5 cells was >50 μM, whereas the selectivity index (SI = CC_50_/EC_50_) for this assay system was >132 for HCoV-OC43 and >94 for HCoV-229E. The EC_50_ of remdesivir against HCoV-OC43 and HCoV-229E was 0.229 ± 0.022 and 0.071 ± 0.005 μM, respectively, which was consistent with a previous report (Brown et al., 2019). To examine the mechanism of HMTU antiviral activity, qRT-PCR analysis measuring both subgenomic (nucleocapsid) and genomic (ORF1b) viral RNA transcripts, a TCID_50_ assay to measure the progeny virus titer, and IFA for the detection of the viral nucleoprotein were performed. qRT-PCR analysis revealed a dose-dependent inhibition of HMTU against HCoV-OC43 (Figure 2A) and HCoV-229E (Figure 2C) replication for both subgenomic (Figures 2A and 2C; blue bars) and genomic (Figures 2A and 2C; red bars) RNA transcripts. The TCID_50_ assay also revealed more than a 1-log reduction in progeny virus titer supernatants of HCoV-OC43-infected Huh7 cells (Figure 2B; dark gray bar) or A549/TMPRSS2 cells (Figure 2B; light gray bar) treated with 1 μM HMTU. Furthermore, IFA demonstrated that positive signals of viral nucleoprotein were also decreased in a dose-dependent fashion after treatment with HMTU (Figure 2D). Thus, these results suggest that HMTU possesses specific antiviral activity against HCoV replication.

**Table 4:** EC_50_ and CC_50_ values of HMTU and remdesivir in HCoV-infected MRC5 cells

Compound	EC_50_ (μM)						CC_50_ (μM)				
	HCoV-OC43			HCoV-229E			MRC5	Huh7	A549/TMPRSS2	VeroE6/TMPRSS2	Caco-2
	Mean	±	SEM	Mean	±	SEM	Mean	Mean	Mean	Mean	Mean
HMTU	0.378	±	0.023	0.528	±	0.029	>50	>20	>20	>20	>20
Remdesivir	0.229	±	0.022	0.071	±	0.005	>50				

Each value represents as the mean ± SEM from three independent experiments.

### HMTU effectively inhibits both SARS-CoV and SARS-CoV-2 replication

**Figure 3:**
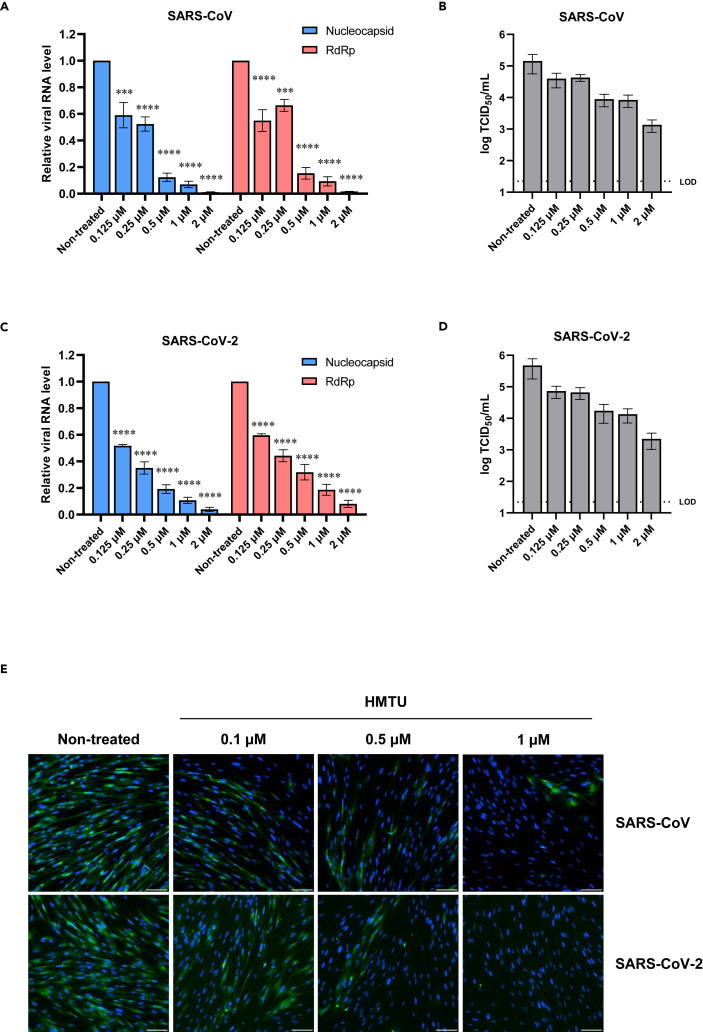
Antiviral activities of HMTU against SARS-CoV and SARS-CoV-2 *in vitro* (A and C) Viral RNA expression was quantified by qRT-PCR analysis. Caco-2 cells were infected with SARS-CoV (A) or SARS-CoV-2 (C) at a multiplicity of infection (MOI) of 0.1 containing a serially diluted compound. At 48 hpi, total RNA was collected, and relative expression of the nucleocapsid and RdRp genes was evaluated by qRT-PCR with β-actin mRNA used as a reference control. Data represent mean values of at least three independent experiments, and error bars indicate SEM. Statistically significant differences were determined using a one-way ANOVA followed by Dunnett's multiple comparisons test; ∗∗∗p < 0.0005 and ∗∗∗∗p < 0.0001. (B and D) Progeny virus titers were quantified by the TCID_50_ assay. Caco-2 cells were infected with SARS-CoV (B) or SARS-CoV-2 (D) at an MOI of 0.1 containing a serially diluted compound. At 48 hpi, supernatants were collected, and dilutions were used to inoculate into VeroE6 or VeroE6/TMPRSS2 cells, respectively. Three days after inoculation, viral titers were determined by calculation of TCID_50_/mL. Data represent mean values of at least three independent experiments, and error bars indicate SEM. Dotted line indicates the LOD. (E) Viral spike protein expression in virus-infected cells. MRC5/ACE2 cells were infected with SARS-CoV at an MOI of 0.1 or SARS-CoV-2 at an MOI of 0.05 containing a serially diluted compound. At 48 hpi, cells were stained with anti-SARS-CoV spike protein antibody (green) and counterstained with Hoechst 33342 nuclear dye (blue). Scale bars indicate 200 μm.

Subsequently, we examined the antiviral activity of HMTU against both SARS-CoV and SARS-CoV-2 using qRT-PCR analysis measuring both subgenomic (nucleocapsid) and genomic (RdRp) viral RNA transcripts, TCID_50_ assays measuring progeny virus titer, and IFA for the detection of the viral spike protein. SARS-CoV (Figure 3A) and SARS-CoV-2 (Figure 3C) RNA replication was inhibited in a dose-dependent manner in both subgenomic (Figures 3A and 3C; blue bars) and genomic (Figures 3A and 3C; red bars) RNA transcripts. Progeny virus titers were reduced by more than 1-log TCID_50_/mL in supernatants of SARS-CoV and SARS-CoV-2-infected cells in the presence of 0.5 μM HMTU (Figures 3B and 3D). Furthermore, inhibition of the viral spike protein expression was dose dependently observed with HMTU. In addition, viral protein expression was significantly inhibited in both SARS-CoV and SARS-CoV-2 in the presence of 1 μM HMTU (Figure 3E). These results suggest that HMTU inhibits the replication of SARS-CoV and SARS-CoV-2 and has a potential for treatment of both viruses.

### HMTU inhibits the post-entry step of the SARS-CoV-2 infection cycle

**Figure 4:**
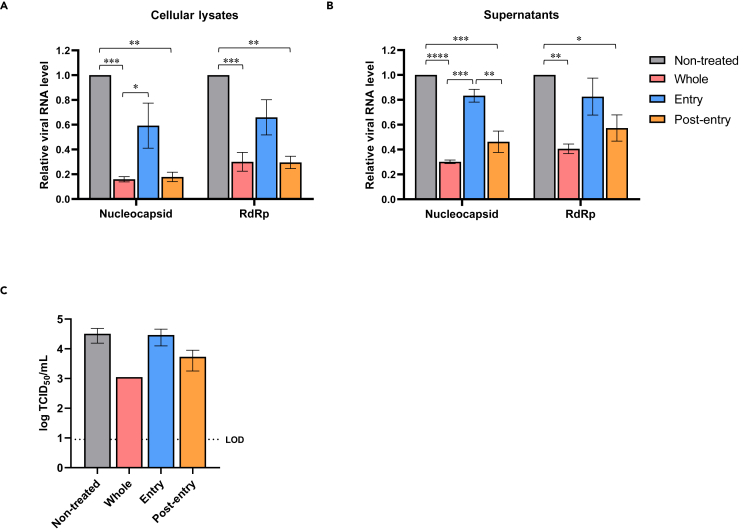
Time-of-addition assay for HMTU against SARS-CoV-2 (A and B) Viral RNA expression was quantified by qRT-PCR analysis. Caco-2 cells were infected with SARS-CoV-2 at a multiplicity of infection (MOI) of 0.1 for 1 h and treated with 1 μM of HMTU at the indicated time points (whole, entry, and post-entry). At 24 hpi, total RNA (A) and supernatants (B) were collected, and relative expressions of the nucleocapsid and RdRp genes were evaluated by qRT-PCR. Intracellular viral RNA levels were normalized to β-actin mRNA. Data represent mean values from two independent experiments, each carried out in triplicates. Error bars indicate SEM. Statistically significant differences were determined using a one-way ANOVA followed by Tukey's multiple comparisons test; ∗p < 0.05, ∗∗p < 0.005, ∗∗∗p < 0.0005, and ∗∗∗∗p < 0.0001. (C) Progeny virus titers were quantified by the TCID_50_ assay. Caco-2 cells were infected with SARS-CoV-2 and treated with HMTU as described above. At 24 hpi, supernatants were collected, and dilutions were used to inoculate VeroE6/TMPRSS2 cells. Three days after inoculation, viral titers were determined by calculation of the TCID_50_/mL. Data represent mean values from two independent experiments, each in triplicates. Error bars indicate SEM. Dotted line indicates the LOD.

Subsequently, we performed a time-of-addition assay following the methods previously described by Wang et al. (Ohashi et al., 2021; Wang et al., 2020). In this assay, Caco-2 cells were treated with 1 μM HMTU at three steps in the SARS-CoV-2 infection cycle (whole, entry, and post-entry) as described in the Methods section, and viral replication was then measured by both qRT-PCR analysis targeting the viral nucleocapsid region and the previously described TCID_50_ assay. qRT-PCR analysis demonstrated that HMTU significantly reduced intracellular and extracellular viral RNAs at the post-entry step but not at the entry step in SARS-CoV-2-infected cells (Figures 4A–4C). These data demonstrated that HMTU contributed to the inhibition of SARS-CoV-2 at a late stage of virus replication.

### HMTU 5′-triphosphate inhibits RNA extension by viral RdRp

Nucleoside analogs are known to inhibit viral RNA synthesis or disrupt viral RNA function. We hypothesized that HMTU-TP could be incorporated into newly synthesized viral RNA by viral RdRp and as a result inhibit viral RNA synthesis. To determine if HMTU-TP is incorporated into the RNA template by viral RdRp, resulting in chain termination, we performed the primer extension assays as previously described by Lu et al. (Lu et al., 2017).

**Figure 5:**
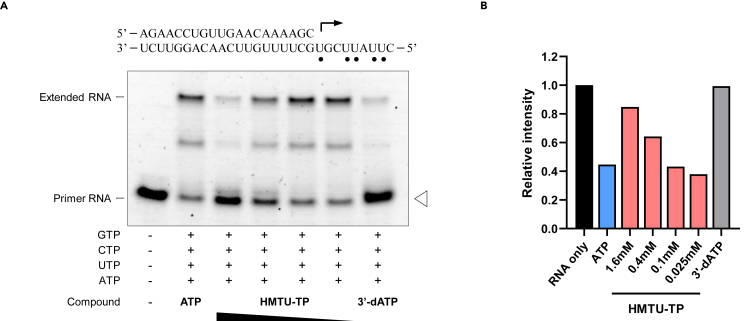
HMTU 5′-triphosphate impairs RNA extension by viral RdRp (A) Analysis of chain termination activity of HMTU 5′-triphosphate (HMTU-TP) by the primer extension assay catalyzed by ZIKV RdRp. The RNA P/T used in this assay is indicated at the top (small black circles indicate the incorporation sites of ATP). ZIKV NS5 and RNA P/T were incubated with ATP analogs (500 μM ATP, 100 μM 3′-dATP, or serially diluted HMTU-TP), as indicated in each lane. Primer extension reactions were initiated by the addition of ATP, GTP, CTP, and UTP (100 μM each). The reactions were performed at 30°C for 2 h and stopped by the addition of quenching buffer. The products were resolved by denaturing PAGE. (B) Relative band intensities of fluorescently labeled RNA primers. Relative fluorescence intensities of each RNA primer (white arrowhead in Figure 5A) were normalized by the RNA sample without ATP or HMTU-TP (black bar, RNA only).

Recombinant ZIKV NS5 protein was incubated with the RNA primer/template (RNA P/T) in the presence of adenosine triphosphate analog [ATP, 3′-deoxyATP (3′-dATP), or HMTU-TP]. Then, reactions were initiated by addition of four natural ribonucleotides (ATP, GTP, CTP, and UTP). As a positive control for chain termination, 3′-dATP was used. As expected, ATP was incorporated into RNA template and caused extension of the template. Incorporation of 3′-dATP prevented RNA extension in the presence of the next correct ribonucleotides (Figure 5A). HMTU-TP was also incorporated into RNA template, resulting in chain termination by ZIKV RdRp (Figures 5A and 5B). These results suggested that HMTU inhibits viral RdRp and synthesis of viral RNA by chain termination.

## Discussion

Many drug-repurposing studies that target SARS-CoV-2 have been carried out, and several candidates and/or urgently approved agents have been employed (Grein et al., 2020; Li and De Clercq, 2020; Ohashi et al., 2021; Riva et al., 2020; Wang et al., 2020). However, in spite of this, no fully effective drugs against SARS-CoV-2 are currently available. In addition other NTDs including DENV, for which there are no fully effective drugs or vaccines require urgent investigation and attention (Mackey and Liang, 2012). Our drug discovery research goals were to identify compounds specific for individual viruses but to also focus on those which might have a broad spectrum of activity against a range of viral families. In this study, we screened our nucleoside analog library and focused on the efficacy of tubercidin derivatives against both flaviviruses and coronaviruses. Our results revealed that the compound HMTU had a broad spectrum of antiviral activity against both flaviviruses, including DENV, ZIKV, YFV, JEV, and WNV, and human coronaviruses, including HCoV-OC43, HCoV-229E, SARS-CoV, and SARS-CoV-2. Since there are no approved therapeutic agents for the treatment of flaviviruses and human coronaviruses, and specifically against the SARS-CoV-2, such drugs are urgently needed. While recently the antiviral activities of 5-substituted tubercidin derivatives have been reported (Milisavljevic et al., 2021), we have here demonstrated that the antiviral activities of differently modified tubercidin analogs were also observed at submicromolar levels (Tables 1, 2, and 3), and we have carried out preliminary studies on both toxicities and on the potential antiviral mechanisms. It is anticipated that further and ongoing studies will help us to better define the latter.

Tubercidin (7-deazaadenosine) and tubercidin derivatives have been reported to exhibit antiviral activities against several viruses such as VSV, coxsackie virus, polio virus, rhinovirus, and ZIKV (Bergstrom et al., 1984; De Clercq et al., 1986; Eyer et al., 2016). Alternatively, it has been known that tubercidin strongly inhibits the proliferation of cells through interfering with numerous cellular processes that can result in significant cytotoxicity (Bergstrom et al., 1984). Therefore, many structure-activity relationship (SAR) studies have been conducted to overcome the latter. Previous studies have achieved successful outcomes with SAR studies, as some deazapurine nucleosides, such as NITD-008 (2′-*C*-ethynyltubercidin) (Yin et al., 2009) and remdesivir (GS-5734, the phosphoramidate prodrug of 1′-cyano 4-aza-7,9-dideazaadenosine *C*-nucleoside analog) (Cho et al., 2012), were tested in nonclinical or clinical trials against various viral infections (Grein et al., 2020; Li and De Clercq, 2020; Yin et al., 2009). Recently, NITD-008 has been reported to demonstrate broad-spectrum antiviral activity against DENV1-4, ZIKV, YFV, WNV, and hepatitis C virus with lower cytotoxicity than tubercidin (Yin et al., 2009). According to previous reports, NITD-008 interferes with viral RdRp activity, leading to an inhibition of DENV2 replication *in vitro* and *in vivo* (Milligan et al., 2018; Yin et al., 2009). However, it exhibited significant toxicity when dosed orally in rats and dogs during the preclinical phases and could therefore not be developed further for clinical trials (Lim et al., 2015; Yin et al., 2009).

Other recent reports have shown that remdesivir may have an anti-Ebolavirus activity, and it has been reported to also have potent antiviral activities against *Filoviridae* (e.g. Marburg virus), *Paramyxoviridae* (e.g. Nipah virus and Hendra virus), *Pneumoviridae* (e.g. respiratory syncytial virus), *Flaviviridae* (e.g. Tick-borne encephalitis virus), and the *Coronaviridae* (e.g. HCoV-OC43, HCoV-229E, SARS-CoV, MERS-CoV, and SARS-CoV-2) (Agostini et al., 2018; Brown et al., 2019; Lo et al., 2017; Sheahan et al., 2017; Wang et al., 2020). Like NITD-008, remdesivir also inhibited viral RdRp activity, and the mechanism of action of remdesivir is considered to be due to delayed chain termination (Gordon et al., 2020; Tchesnokov et al., 2020). RdRp on the other hand is indispensable for the replication and transcription of the viral genome. Thus while there is a divergence of RdRp sequences between each viral family, the core structural features of RdRps are conserved (Venkataraman et al., 2018). These findings suggest that viral RdRp inhibitors, including nucleoside analogs, have the potential to serve as effective broad-spectrum antivirals.

HMTU is also a 7-deazaadenosine analog and C-5 substituted derivative of tubercidin. Although tubercidin and its C-5 substitutes have been reported to be cytotoxic adenosine analogs (Bergstrom et al., 1984), no strong cytotoxicity of HMTU was observed in our studies (Tables 1 and 4). Our study also revealed that HMTU inhibited both flavivirus and human coronavirus infections and dose dependently inhibited viral RNA replication, viral protein expression, and progeny virus production of HCoVs, SARS-CoV, and SARS-CoV-2 by functioning at a late stage of viral replication (Tables 2 and 3 and Figures 2, 3, and 4). HMTU was also found to potently inhibit both subgenomic and genomic viral RNA replication in coronavirus-infected cells and inhibited RNA extension after incorporation by viral RdRp. Our findings suggest that HMTU has the potential to be a lead compound for the development of a broad spectrum of anti-flavivirus and anti-coronavirus agents, including SARS-CoV-2, and that these could thus be further used for new drug discovery against SARS-CoV-2.

### Limitations of the study

Herein, we have using several approaches demonstrated the antiviral activities of tubercidin derivatives against both flaviviruses and human coronaviruses *in vitro* and have shown that HMTU had potent antiviral activity against both flaviviruses and coronaviruses, including SARS-CoV-2, and that this did not show cytotoxicity in several cell lines. However, *in vivo* antiviral activities and *in vivo* kinetics studies were not conducted in this study. Thus, further studies are needed to assess its safety, effectiveness, and potential clinical applications.

## STAR★Methods

### Key resources table

**Table:** 

Reagent or Resource	Source	Identifier
**Antibodies**		

Mouse monoclonal anti-coronavirus antibody	Merck Millipore	Cat# MAB9013; RRID: AB_95425
Mouse monoclonal SARS-CoV / SARS-CoV-2 Spike antibody [1A9]	GeneTex	Cat# GTX632604; RRID: AB_2864418
Goat anti-Mouse IgG (H+L) Highly Cross-Adsorbed Secondary Antibody, Alexa Fluor Plus 488	Invitrogen	Cat# A32723; RRID: AB_2633275
Hoechst 33342, Trihydrochloride, Trihydrate - FluoroPure Grade	Molecular Probes	Cat# H21492

**Bacterial and virus strains**		

Dengue virus 1 (D1/hu/PHL/10–07)	National Institute of Infectious Diseases	N/A
Dengue virus 2 (D2/hu/INDIA/09–74)	National Institute of Infectious Diseases	GenBank: LC367234
Dengue virus 3 (D3/hu/Thailand/00–40)	National Institute of Infectious Diseases	N/A
Dengue virus 4 (D4/hu/Solomon/09–11)	National Institute of Infectious Diseases	N/A
Zika virus (MR766)	National Institute of Infectious Diseases	GenBank: LC002520
Yellow fever virus (17D–204)	Nagasaki University	N/A
Japanese encephalitis virus (Beijing–1)	Hokkaido University	N/A
West Nile virus (NY99)	Hokkaido University	N/A
Human coronavirus OC43	ATCC	Cat# VR-1558
Human coronavirus 229E	ATCC	Cat# VR-740
SARS-CoV (Hanoi)	Nagasaki University (Thai et al., 2004)	N/A
SARS-CoV-2 (JP/TY/WK-521)	National Institute of Infectious Diseases (Matsuyama et al., 2020)	GISAID: EPI_ISL_408667

**Chemicals, peptides, and recombinant proteins**		

5-hydroxymethyltubercidin (HMTU)	This paper	N/A
HMTU 5’-triphosphate (HMTU-TP)	This paper	N/A
Ribavirin	FUJIFILM Wako	Cat# 188-02333
Favipiravir	PharmaBlock Sciences	N/A
Remdesivir (GS-5734)	MedChemExpress	Cat# HY-104077
ATP solution	Thermo Fisher Scientific	Cat# R0441
3'-Deoxyadenosine-5'-Triphosphate (3’-dATP)	TriLink BioTechnologies	Cat# N-3001
Recombinant ZIKV NS5 protein	Sino Biological	Cat# 40546-V08B
RNasin Ribonuclease inhibitor	Promega	Cat# N2115

**Critical commercial assays**		

PureLink RNA Mini Kit	Invitrogen	Cat# 12183025
PureLink Pro 96 Viral RNA/DNA Kit	Invitrogen	Cat# 12280096A
THUNDERBIRD Probe One-step qRT–PCR kit	TOYOBO	Cat# QRZ-101
Novex TBE-Urea Gels, 15%	Invitrogen	Cat# EC68852BOX

**Experimental models: Cell lines**		

Hamster: BHK-21 cells	ATCC	CCL-10
African green monkey: Vero E6 cells	ATCC	CRL-1586
Human: MRC5 cells	ATCC	CCL-171
Human: 293T cells	RIKEN BRC	RCB2202
Human: Huh7 cells	RIKEN BRC	RBRC-RCB1942
Human: A549 cells	RIKEN BRC	RCB0098
Human: Caco-2 cells	RIKEN BRC	RCB0988
Human TMPRSS2-expressing Vero E6 cells	Sasaki et al., 2021	N/A
Human TMPRSS2-expressing A549 cells	This paper	N/A
Human ACE2-expressing MRC5 cells	Uemura et al., 2021	N/A

**Oligonucleotides**		

FAM-labeled RNA primer: 5'-56FAM/rArGrArAr CrCrUrGrUrUrGrArArCrArArArArGrC-3'	This paper	N/A
RNA template: 5'-rCrUrUrArUrUrCrGrUrGrCrUr UrUrUrGrUrUrCrArArCrArGrGrUrUrCrU-3'	This paper	N/A
ACTB (Hs01060665_g1)	Applied Biosystems	Cat# 4351368

**Recombinant DNA**		

CSII-CMV-TMPRSS2-IRES2-Bsd	Sasaki et al., 2021	N/A
pLVSIN-CMV-Puro-ACE2	Uemura et al., 2021	N/A

**Software and algorithms**		

GraphPad Prism version 8.4.2	GraphPad Software	https://www.mdf-soft.com
QuantStudio 7 Flex Real-Time PCR System	Applied Biosystems	https://www.thermofisher.com
QuantStudio 7 Flex Real-Time PCR System Software	Applied Biosystems	https://www.thermofisher.com
Fluorescence microscopy, IX73	Olympus	https://www.olympus-lifescience.com
cellSens Standard 1.16	Olympus	https://www.olympus-lifescience.com
Amersham ImageQuant 800 Fluor system	Cytiva	https://www.cytivalifesciences.com
ImageQuant TL version 8.2.0	Cytiva	https://www.cytivalifesciences.com

### Resource availability

#### Lead contact

Further information and requests for resources and reagents should be directed to and will be fulfilled by the lead contact, Akihiko Sato: akihiko.sato@shionogi.co.jp.

#### Materials availability

This study did not generate new unique materials.

### Experimental model and subject details

#### Cell lines

BHK-21 (ATCC), 293T (RIKEN BRC), VeroE6 (ATCC), Huh7 (RIKEN BRC), and A549 (RIKEN BRC) cells were maintained in high-glucose Dulbecco’s modified Eagle’s medium (Gibco; Thermo Fisher Scientific) supplemented with 10% fetal bovine serum (FBS, Gibco) and penicillin–streptomycin (P/S, Wako) at 37°C. MRC5 (ATCC) and Caco-2 cells (RIKEN BRC) were also maintained in Minimum Essential Medium GlutaMAX Supplement (Gibco) supplemented with 10% FBS, nonessential amino acids (Wako), sodium pyruvate (Wako), and P/S at 37°C.

#### Viruses

DENV1 (D1/hu/PHL/10–07), DENV2 (D2/hu/INDIA/09–74), DENV3 (D3/hu/Thailand/00–40), DENV4 (D4/hu/Solomon/09–11), ZIKV (MR766), YFV (17D–204), JEV (Beijing–1), and WNV (NY99) were propagated as described in previous reports (Nobori et al., 2018).

Human coronavirus strain OC43 (HCoV-OC43) and 229E (HCoV-229E) were purchased from ATCC (VR-1558 and VR-740, respectively) and amplified on MRC5 cells. SARS-CoV strain Hanoi was kindly provided by Dr. Kouichi Morita (Nagasaki University, Nagasaki, Japan) and was amplified in VeroE6 cells (Thai et al., 2004), whereas the SARS-CoV-2 strain JPN/TY/WK–521, a clinical isolate from a patient with COVID-19 (Matsuyama et al., 2020) was kindly provided by Dr. Masayuki Shimojima (National Institute of Infectious Diseases, Tokyo, Japan) and amplified on VeroE6/TMPRSS2 cells. WNV, SARS-CoV, and SARS-CoV-2 were propagated in a biosafety level-3 (BSL-3).

Virus infectious titers were also measured by inoculating respective cells with serial dilutions of the virus; while the cytopathic effect (CPE) was scored to calculate the TCID_50_/mL.

### Method details

#### Generation of TMPRSS2- and ACE2-expressing Cells

VeroE6 and A549 cells stably expressing human TMPRSS2 (VeroE6/TMPRSS2 and A549/TMPRSS2) were generated by lentiviral transduction with CSII-CMV-TMPRSS2-IRES2-Bsd (Sasaki et al., 2021) and blasticidin-based selection. MRC5 cells stably expressing human ACE2 (MRC5/ACE2) were also generated by lentiviral transduction as described in previous report (Uemura et al., 2021). For the lentiviral vector preparation, 293T cells were co-transfected with the aforementioned lentiviral vector plasmid and Lentiviral High Titer Packaging Mix (Takara Bio, Shiga, Japan).

#### Compounds

All tubercidin derivatives, including 5-hydroxymethyltubercidin (HMTU) (Bergstrom et al., 1981; Uematsu and Suhadolnik, 1973; Watanabe and Ueda, 1983), were synthesized at the Faculty of Pharmaceutical Sciences, Hokkaido University and their chemical identity and purity were determined by high-performance liquid chromatography and mass spectrometry analysis. Ribavirin, Favipiravir, and GS-5734 (Remdesivir) were purchased from FUJIFILM Wako Pure Chemical Corporation, PharmaBlock Sciences, Inc. and MedChemExpress, respectively, after which all compounds were solubilized in 100% dimethyl sulfoxide (DMSO; Sigma-Aldrich) for *in vitro* studies.

For the RNA extension assays, HMTU 5’-triphosphate (HMTU-TP) was synthesized at the Graduate School of Pharmaceutical Sciences, Tokushima University and their chemical identity and purity were determined by nuclear magnetic resonance and mass spectrometry analysis (Figure S1 and Data S1). HMTU-TP was solubilized in nuclease-free water. ATP and 3’-dATP were purchased from Thermo Fisher Scientific and TriLink BioTechnologies.

#### Cytopathic effect-based antiviral and cytotoxicity assays

The MTT (3-[4,5-dimethyl-2-thiazolyl]-2,5-diphenyl-2H-tetrazolium bromide) and resazurin reduction assays were carried out as previously described (Nobori et al., 2018; Sanaki et al., 2019). These were performed to calculate cell viability following viral induced CPE or cytotoxicity. Assay conditions for flaviviruses are as described in Table 2.

For human coronavirus CPE-based antiviral assay, MRC5 cells were seeded onto 96-well plates with serially diluted compounds in each well. The cells were infected with HCoV-OC43 or HCoV-229E at a multiplicity of infection (MOI) of 0.05 or 0.01, respectively. After 72 h at 37°C, the resazurin reduction assay was conducted to calculate cell viability.

The EC_50_ value was defined in GraphPad Prism version 8.4.2 (GraphPad Software) with a variable slope (four parameters). Noninfected cells were used as a control for 100% inhibition, whereas for infected cells, DMSO alone was used as a control for 0% inhibition. The CC_50_ value for each cell line was also measured using the same method. Cell-free samples were used as 100% cytotoxicity control and DMSO-treated cells were used as 0% cytotoxicity control.

#### Quantification of viral RNA with real-time quantitative reverse transcription PCR (qRT–PCR)

For HCoV-OC43 and HCoV-229E, Huh7 cells were seeded the previous day and infected with HCoV-OC43 at an MOI of 0.1 or HCoV-229E at an MOI of 0.05 containing the serially diluted compound for 1 h. For SARS-CoV and SARS-CoV-2, Caco-2 cells were seeded the previous day and also infected with these viruses at an MOI of 0.1 containing the serially diluted compound for 1 h. After incubation, the unbound virus was removed and new medium containing the serially diluted compound was added. At 48 h post-infection (hpi), total RNA was isolated with PureLink RNA Mini Kit (Invitrogen; Thermo Fischer Scientific).

For rescreening of the nine tubercidin derivatives, Caco-2 cells were seeded the previous day and infected with SARS-CoV-2 at an MOI of 0.1 containing the serially diluted compound for 48 h. After incubation, the culture supernatants were collected, after which viral RNA was isolated by PureLink Pro 96 Viral RNA/DNA Kit (Invitrogen; Thermo Fischer Scientific).

Viral RNA from all samples was quantified using real-time RT–PCR analysis with THUNDERBIRD Probe One-step qRT–PCR kit (TOYOBO) and QuantStudio 7 Flex Real-Time PCR system (Applied Biosystems; Thermo Fischer Scientific). The primers and probe sequences (Integrated DNA Technologies, IDT) targeting the nucleocapsid gene to detect subgenomic viral RNA and the ORF1b or RdRp gene to detect viral genomic RNA were designed in previous reports (Brown et al., 2019; Corman et al., 2020; Emery et al., 2004; Loens et al., 2012), with the primers and probe for ACTB (Hs01060665_g1, Applied Biosystems) transcripts used as internal controls.

#### Virus replication assay (TCID_50_ Assay)

For testing samples were collected using the same method as in the qRT–PCR assays. At 48 hpi, culture supernatants were also collected, after which, serial dilutions were prepared. Dilutions were used to inoculate MRC5 cells for HCoVs, VeroE6 cells for SARS-CoV, and VeroE6/TMPRSS2 cells for SARS-CoV-2, and at 72 hpi, viral titers were determined by calculating the TCID_50_/mL.

#### Indirect immunofluorescence assay

For HCoV-OC43, A549/TMPRSS2 cells were seeded on the day before viral infection, after which, cells were infected with HCoV-OC43 at an MOI of 1 containing the serially diluted compound. For SARS-CoV and SARS-CoV-2, MRC5/ACE2 cells were seeded on the day before viral infection, after which, cells were infected with SARS-CoV at an MOI of 0.1 or SARS-CoV-2 at an MOI of 0.05 containing the serially diluted compound. At 48 hpi, cells were fixed with 4% paraformaldehyde (Nacalai tesque) or the Masked Form A (Japan Tanner Co.), permeabilized with 0.5% Triton X-100 in PBS or ice-cold methanol. Cells were then stained with the anti-coronavirus antibody (MAB9013, Merck Millipore), anti-SARS-CoV Spike antibody (1A9; GTX632604, GeneTex) and Alexa Fluor Plus 488-conjugated anti-mouse IgG antibody (Invitrogen). Cell nuclei were counterstained with Hoechst 33342 (Molecular Probes; Thermo Fischer Scientific). Cells were then evaluated using fluorescence microscopy (IX73, Olympus). Images were processed with cellSens Standard 1.16 (Olympus).

#### Time-of-addition assays

The time-of-addition assay was performed according to previous reports (Ohashi et al., 2021; Wang et al., 2020). Briefly, Caco-2 cells were seeded on the day before SARS-CoV-2 infection (MOI of 0.1) and compound treatment. One micromolar HMTU or DMSO was then added at the indicated time points (Whole, Entry, and Post-entry). For “Whole” treatment, the cells were incubated with both the compound and virus for 1 h. After incubation, unbound viruses were removed, and the cells were cultured with HMTU-containing medium for 2 h to allow viral entry. At 3 hpi, culture supernatants were replaced with new HMTU-containing media and maintained until the end of the experiment. For “Entry” treatment, the cells were incubated as described above, and at 3 hpi, HMTU-containing media were replaced with fresh culture media and maintained until the end of the experiments. For “Post-entry” treatment, HMTU was added to the virus-infected cells at 3 hpi and maintained until the end of the experiment. At 24 hpi, total RNA was isolated, and viral RNA was quantified using qRT–PCR analysis as described above. Supernatants were collected, after which serial dilutions of these supernatants were used to inoculate a monolayer of the VeroE6/TMPRSS2 cells. Three days after inoculation, the CPE was scored, and TCID_50_/mL was calculated to measure viral titers.

#### Primer and template annealing

To generate RNA primer-template complexes (RNA P/T), 0.5 μM fluorescently labeled RNA primer and 2 μM unlabeled RNA template were mixed in 50 mM NaCl, incubated at 95°C for 10 min, and then slowly cooled down to room temperature. The annealed RNA P/T was stored at -30°C before use in the primer extension assay.

#### Primer extension polymerase activity assay

The primer extension assay was performed according to a previous report (Lu et al., 2017). For analysis of the competitive inhibition ability of HMTU-TP, 200 nM of recombinant ZIKV NS5 (Sino Biological) and 50 nM RNA P/T were incubated 30°C for 15 min in reaction buffer [10 mM Tris-HCl (pH 7.5), 10 mM DTT, 5 mM MgCl_2_, 5% glycerol, 0.05% Triton-X 100, and 0.02 U/μL RNasein (Promega)], and then ATP analogs [500 μM ATP (Thermo Fischer Scientific), 100 μM 3’-dATP (TriLink), or serially diluted HMTU-TP (0.025, 0.1, 0.4 or 1.6 mM)] were added and incubated at 30°C for 10 min. Primer extension reactions were initiated by the addition of ATP, GTP, CTP and UTP (100 μM each). The reactions were performed 30°C for 2 h and stopped by the addition of quenching buffer (7M Urea, 1X TBE buffer, and 50 mM EDTA). The quenched samples were denatured at 95°C for 5 min, and the primer extension products were separated on a 15% denaturing polyacrylamide gel (Invitrogen). After electrophoresis, the gels were scanned using an Amersham ImageQuant 800 Fluor system (Cytiva). The band intensities were analyzed by ImageQuant TL version 8.2.0 (Cytiva).

### Quantification and statistical analysis

All bar graphs in this study were presented as mean ± SEM. One-way ANOVA followed by Dunnett’s or Tukey’s multiple comparisons test was also performed to determine the statistical significance using GraphPad Prism version 8.4.2. A p value of < 0.05 was considered statistically significant; ∗ p < 0.05, ∗∗ p < 0.005, ∗∗∗ p < 0.0005, and ∗∗∗∗ p < 0.0001.

## Data Availability

This study did not generate and analyze any datasets or code. All data are included in the article and supplemental information and any additional information will be available from the lead contact upon request.
