# Supplementary material for: 5-Hydroxymethyltubercidin exhibits potent antiviral activity against flaviviruses and coronaviruses, including SARS-CoV-2
Source: iScience. 2021 Sep 11;24(10):103120. doi: 10.1016/j.isci.2021.103120 (PMC8433052; doi:10.1016/j.isci.2021.103120)
Supplement: Document S1. Figure S1 and Data S1 [file mmc1.pdf]

iScience, Volume *24*

Supplemental information

5-Hydroxymethyltubercidin exhibits potent antiviral activity against flaviviruses and coronaviruses, including SARS-CoV-2

Kentaro Uemura, Haruaki Nobori, Akihiko Sato, Takao Sanaki, Shinsuke Toba, Michihito Sasaki, Akiho Murai, Noriko Saito-Tarashima, Noriaki Minakawa, Yasuko Orba, Hiroaki Kariwa, William W. Hall, Hirofumi Sawa, Akira Matsuda, and Katsumi Maenaka

Figure S1. Synthesis and characterization of HMTU-TP, Related to STAR Methods

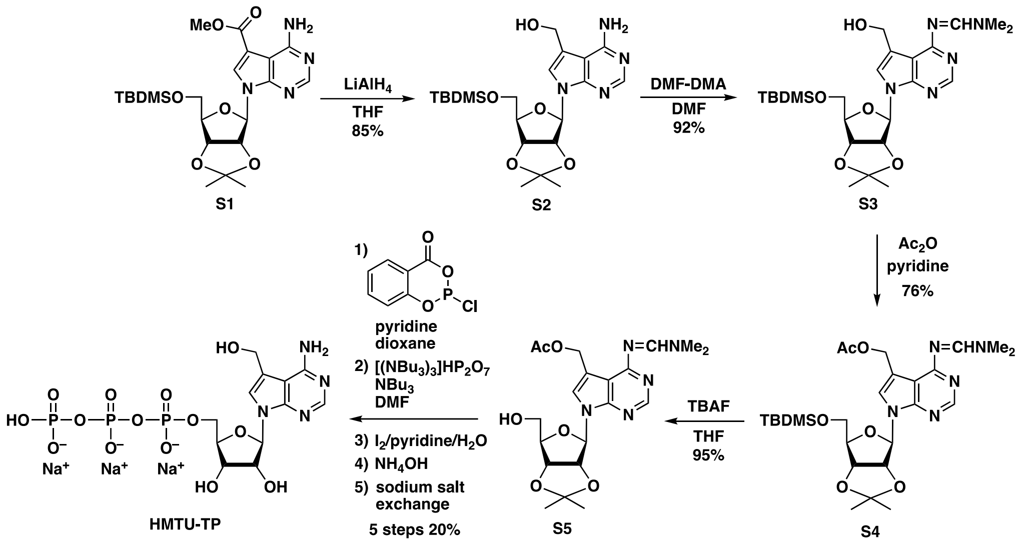

Data S1. List of NMR spectra, Related to STAR Methods

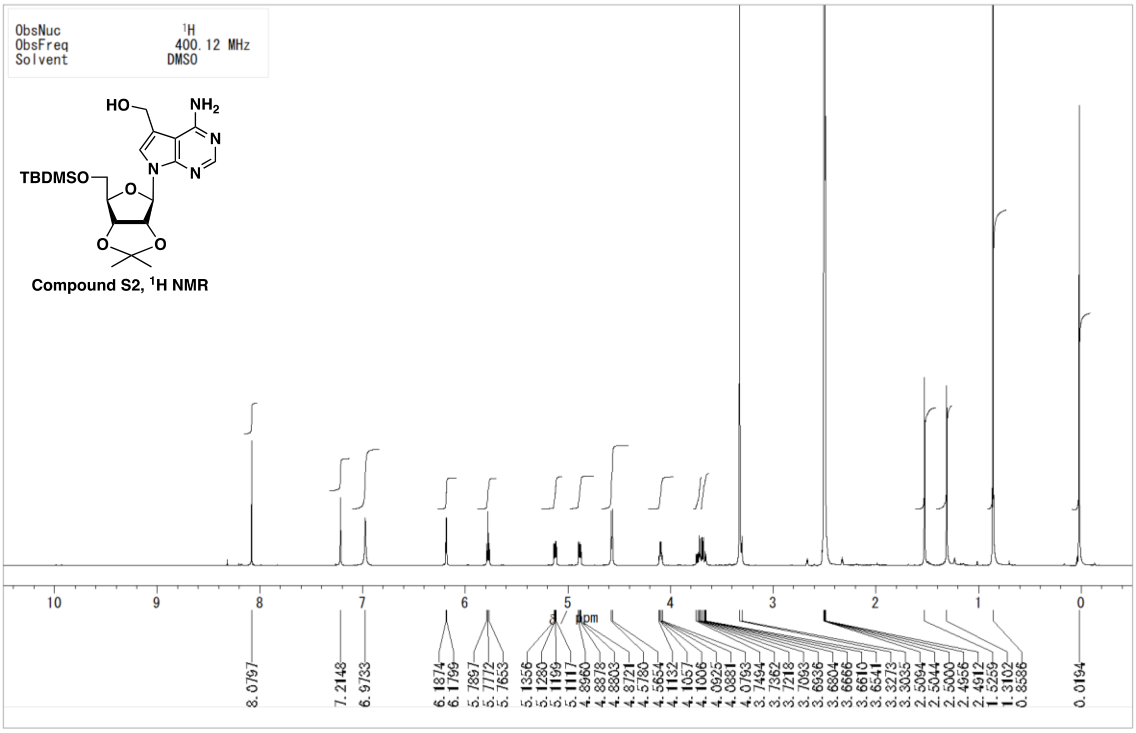

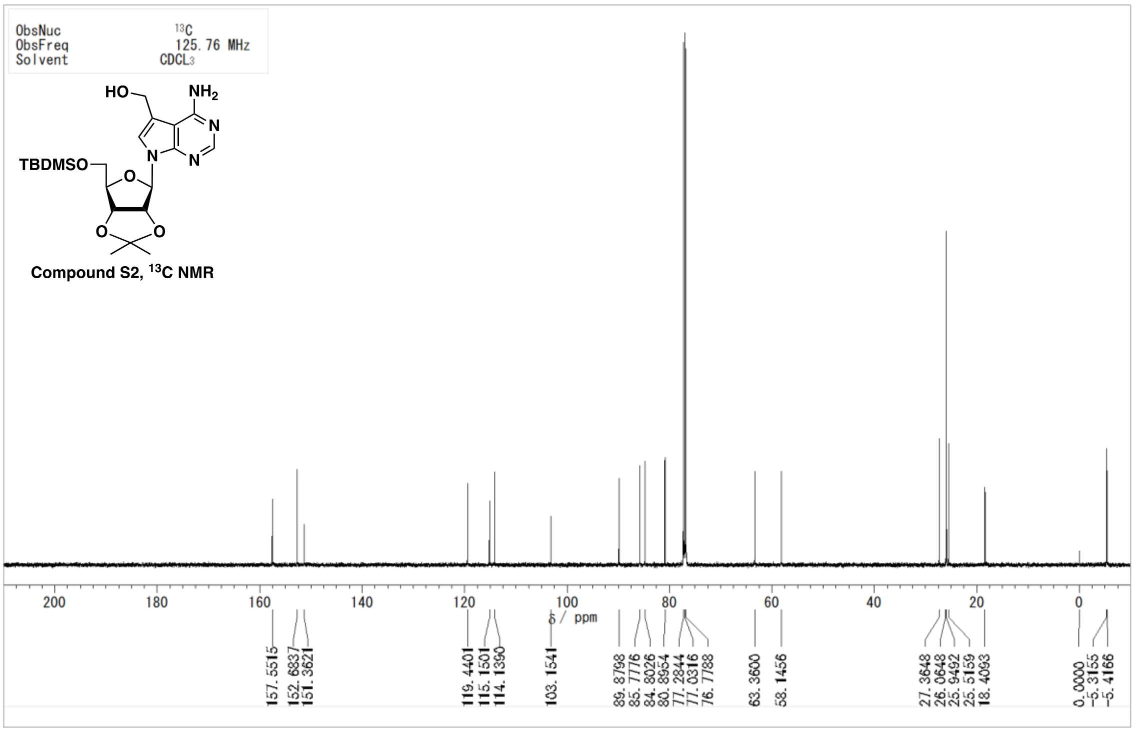
ObsNuc 13C
ObsFreq 125.76 MHz
Solvent CDCL3
HO
NH2
N
N
N
TBDMSO
O
O
O
Compound S2, 13C NMR
200
180
160
140
120
100
80
60
40
20
0
δ / ppm
157.5515
152.6837
151.3621
119.4401
115.1501
114.1390
103.1541
89.8798
85.7776
84.8026
80.8954
77.2844
77.0316
76.7788
63.3600
58.1456
27.3648
26.0648
25.9492
25.5159
18.4093
0.0000
-5.3155
-5.4166
6

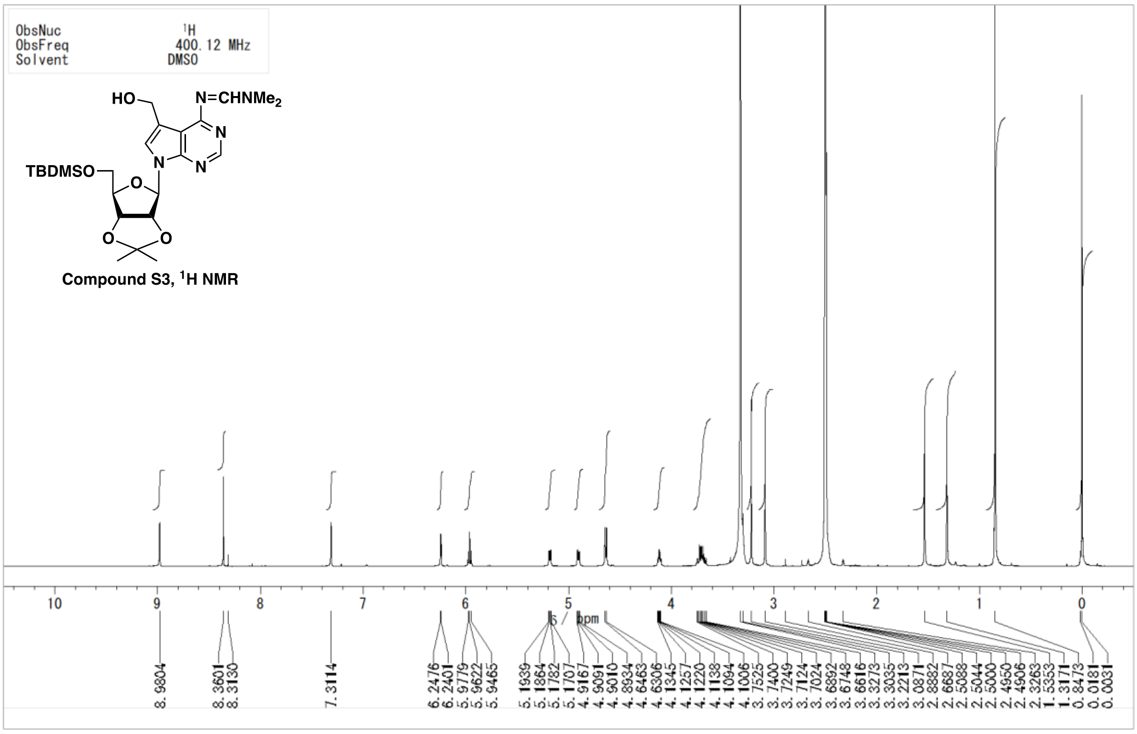
ObsNuc 1H
ObsFreq 400.12 MHz
Solvent DMSO
HO
N=CHNMe2
N
N
N
TBDMSO
O
O
O
Compound S3, 1H NMR
10
9
8
7
6
5
4
3
2
1
0
δ / ppm
8.9804
8.3601
8.3130
7.3114
6.2476
6.2401
5.9779
5.9622
5.9465
5.1939
5.1864
5.1782
5.1707
4.9167
4.9091
4.9010
4.8934
4.6463
4.6306
4.1345
4.1257
4.1220
4.1138
4.1094
4.1006
3.7525
3.7400
3.7249
3.7124
3.7024
3.6892
3.6748
3.6616
3.3273
3.3035
3.2213
3.0871
2.8882
2.6687
2.5088
2.5044
2.5000
2.4950
2.4906
2.3263
1.5353
1.3171
0.8473
0.0181
0.0031
7

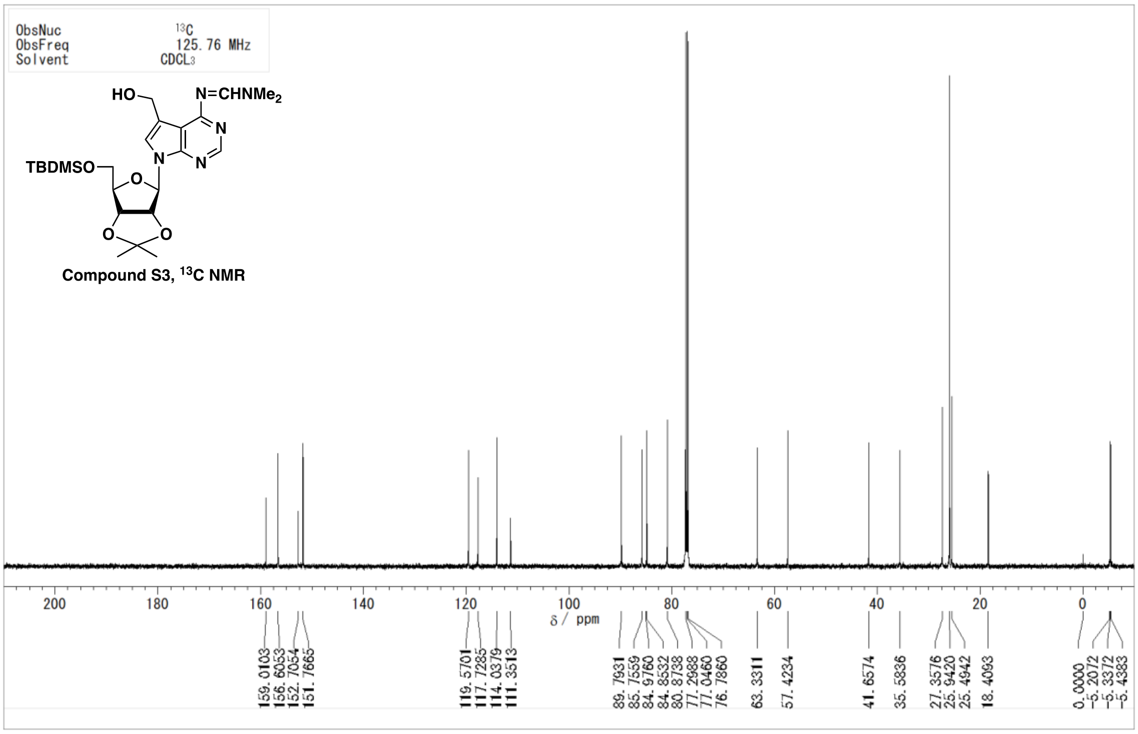

ObsNuc 13C
ObsFreq 125.76 MHz
Solvent CDCL3
HO
N=CHNMe2
N
N
N
TBDMSO
O
O
O
Compound S3, 13C NMR
200
180
160
140
120
100
δ / ppm
80
60
40
20
0
159.0103
156.6053
152.7054
151.7665
119.5701
117.7285
114.0379
111.3513
89.7931
85.7559
84.9760
84.8532
80.8738
77.2988
77.0460
76.7860
63.3311
57.4234
41.6574
35.5836
27.3576
25.9420
25.4942
18.4093
0.0000
-5.2072
-5.3372
-5.4383

8

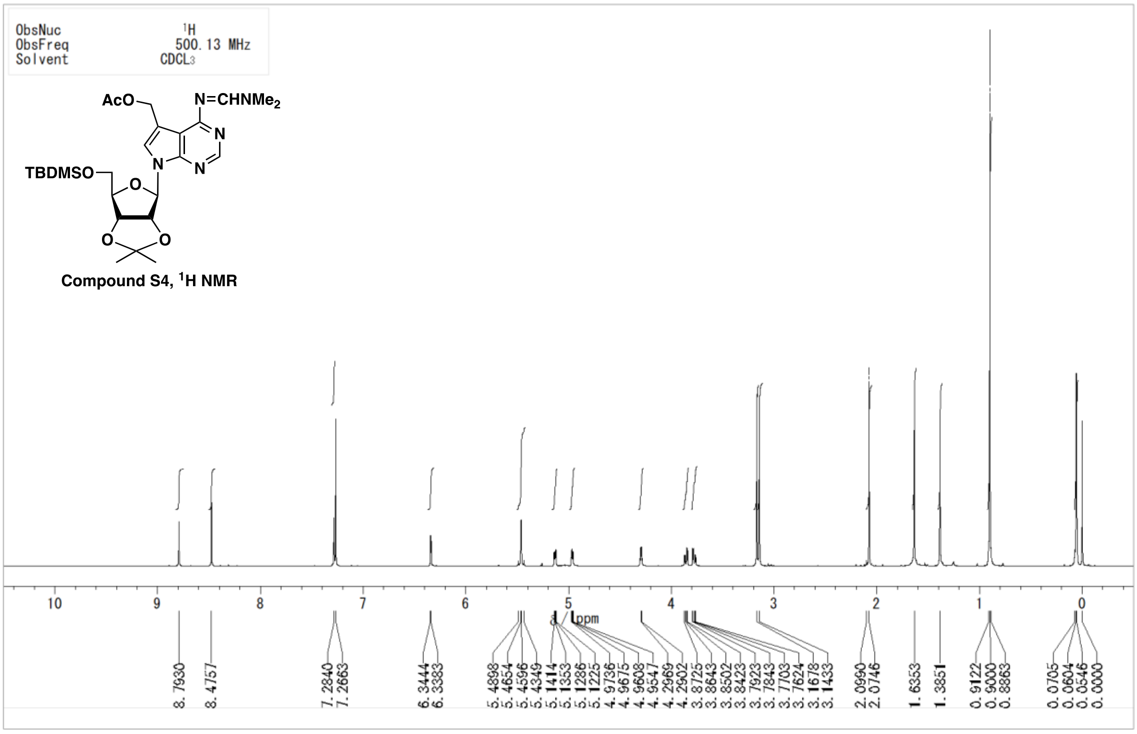

ObsNuc 1H
ObsFreq 500.13 MHz
Solvent CDCL3
AcO
N=CHNMe2
N
N
N
TBDMSO
O
O
O
Compound S4, 1H NMR
10
9
8
7
6
5
4
3
2
1
0
δ / ppm
8.7930
8.4757
7.2840
7.2663
6.3444
6.3383
5.4898
5.4654
5.4596
5.4349
5.1414
5.1353
5.1286
5.1225
4.9736
4.9675
4.9608
4.9547
4.2969
4.2902
3.8725
3.8643
3.8502
3.8423
3.7923
3.7843
3.7703
3.7624
3.1678
3.1433
2.0990
2.0746
1.6353
1.3851
0.9122
0.9000
0.8863
0.0705
0.0604
0.0546
0.0000

9

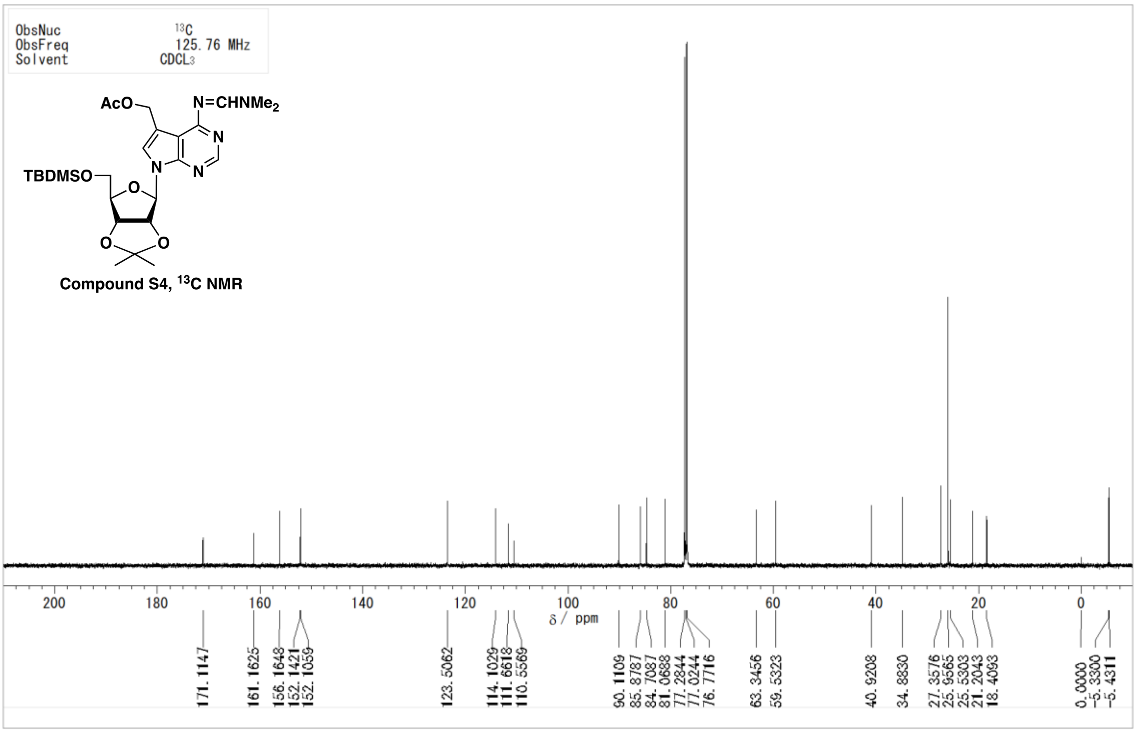
ObsNuc 13C
ObsFreq 125.76 MHz
Solvent CDCL3
AcO
N=CHNMe2
N
N
N
TBDMSO
O
O
O
Compound S4, 13C NMR
δ / ppm
171.1147
161.1625
156.1648
152.1421
152.1059
123.5062
114.1029
111.6618
110.5569
90.1109
85.8787
84.7087
81.0688
77.2844
77.0244
76.7716
63.3456
59.5323
40.9208
34.8830
27.3576
25.9565
25.5303
21.2043
18.4093
0.0000
-5.3300
-5.4311

10

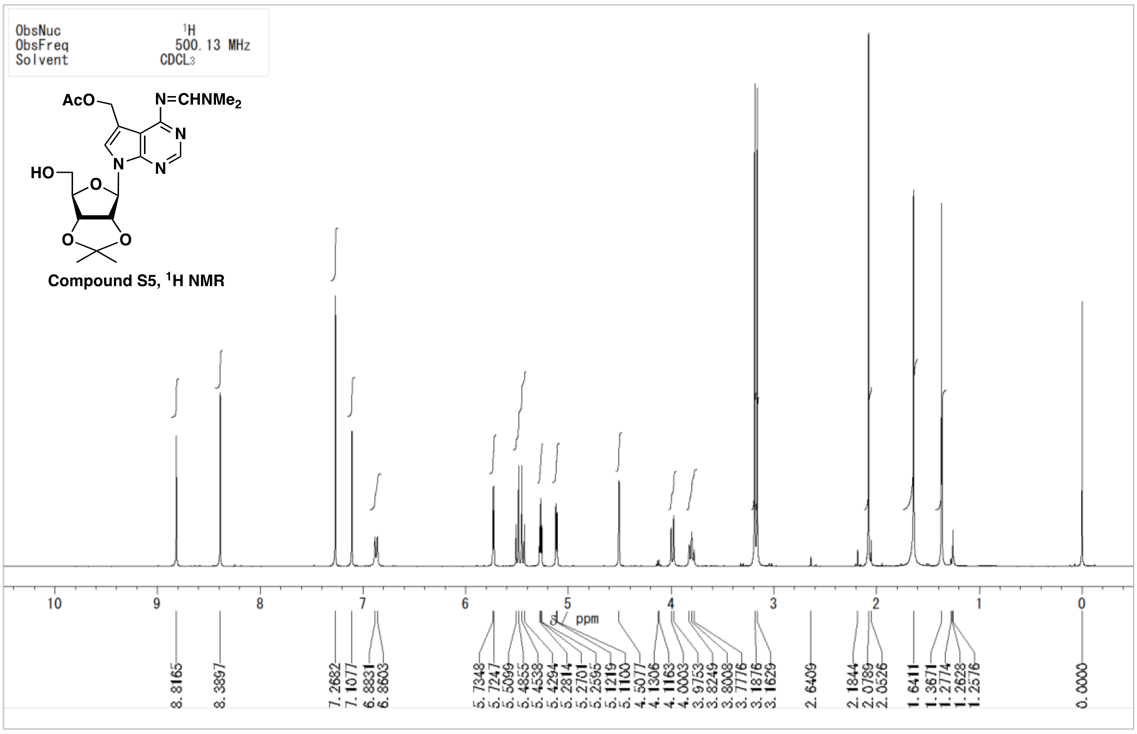
ObsNuc 1H
ObsFreq 500.13 MHz
Solvent CDCL3
AcO
N=CHNMe2
N
N
N
HO
O
O
O
Compound S5, 1H NMR
δ / ppm
8.8165
8.3897
7.2682
7.1077
6.8831
6.8603
5.7348
5.7247
5.5099
5.4855
5.4538
5.4294
5.2814
5.2701
5.2595
5.1219
5.1100
4.5077
4.1306
4.1163
4.0003
3.9753
3.8249
3.8008
3.7776
3.1876
3.1629
2.6409
2.1844
2.0789
2.0526
1.6411
1.3671
1.2774
1.2628
1.2576
0.0000

11

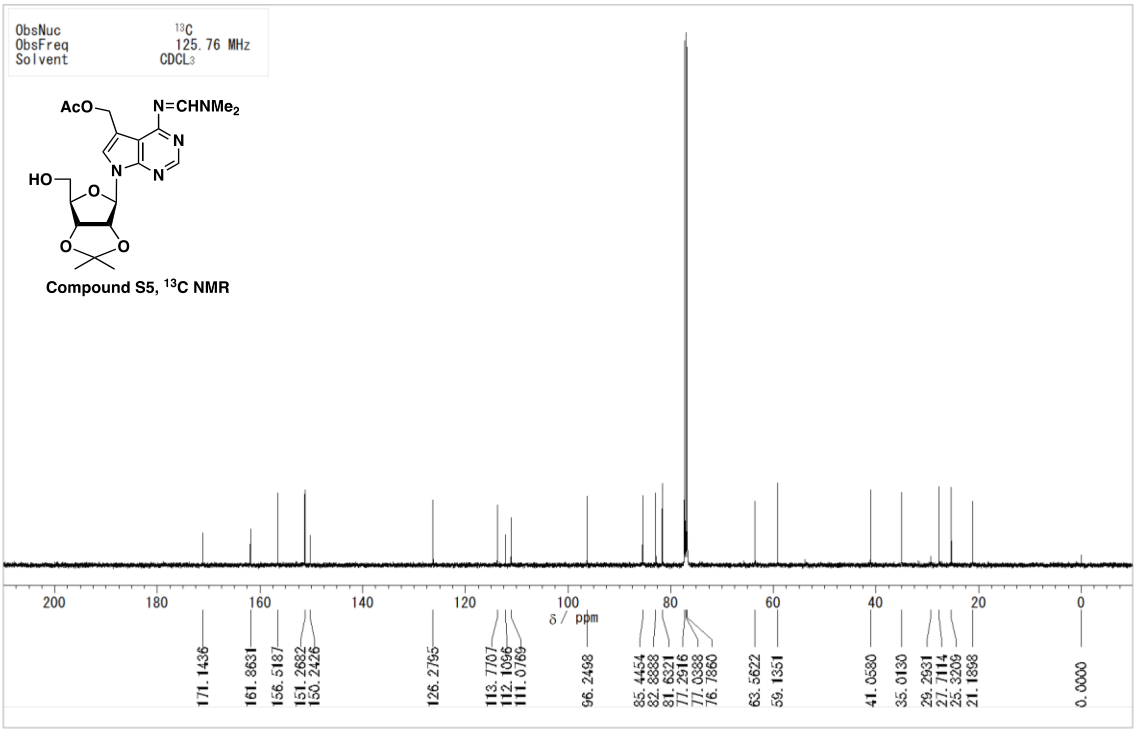
ObsNuc ^{13}C
ObsFreq 125.76 MHz
Solvent CDCL3
AcO
N=CHNMe2
N
N
N
HO
O
O
O
Compound S5, ^{13}C NMR
δ / ppm
171.1436
161.8631
156.5187
151.2682
150.2426
126.2795
113.7707
112.1096
111.0769
96.2498
85.4454
82.8888
81.6321
77.2916
77.0388
76.7860
63.5622
59.1351
41.0580
35.0130
29.2931
27.7114
25.3209
21.1898
0.0000

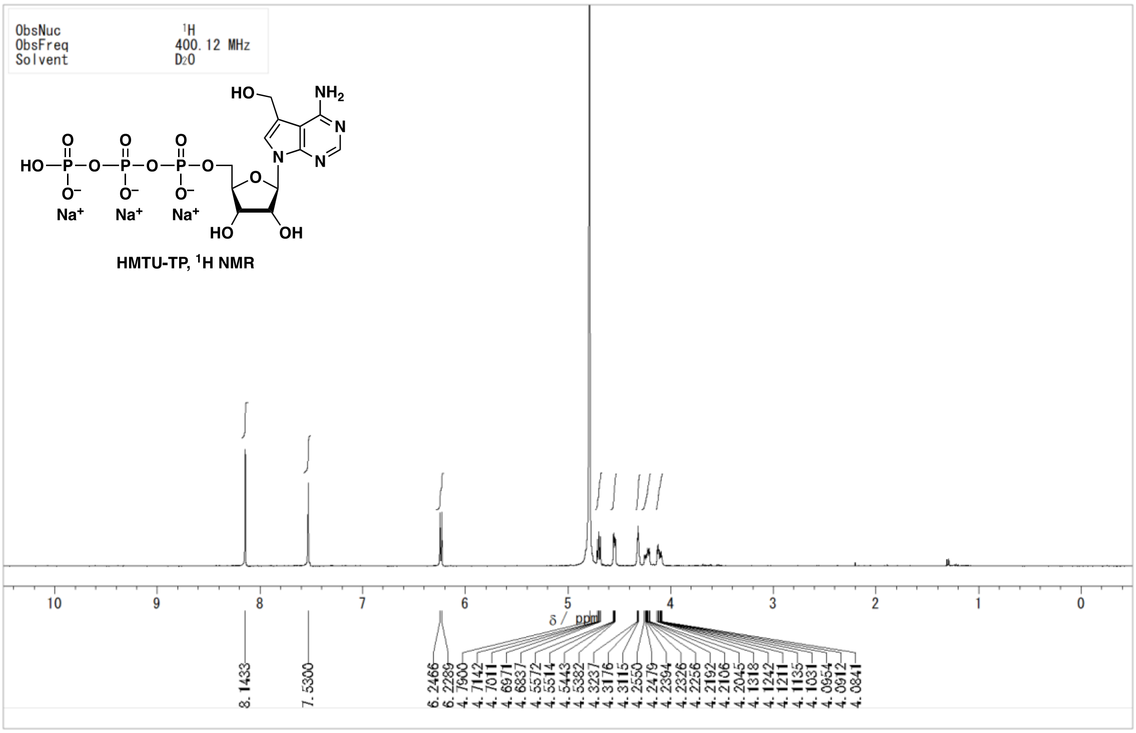
ObsNuc ^{1}H
ObsFreq 400.12 MHz
Solvent D2O
HO
NH2
N
N
N
O
HO–P–O–P–O–P–O
O⁻ O⁻ O⁻
Na+ Na+ Na+
HO OH
HMTU-TP, ^{1}H NMR
δ / ppm
8.1433
7.5300
6.2466
6.2289
4.7900
4.7142
4.7011
4.6971
4.6837
4.5572
4.5514
4.5443
4.5382
4.3237
4.3176
4.3115
4.2550
4.2479
4.2394
4.2326
4.2256
4.2192
4.2106
4.2045
4.1318
4.1242
4.1211
4.1135
4.1031
4.0954
4.0912
4.0841

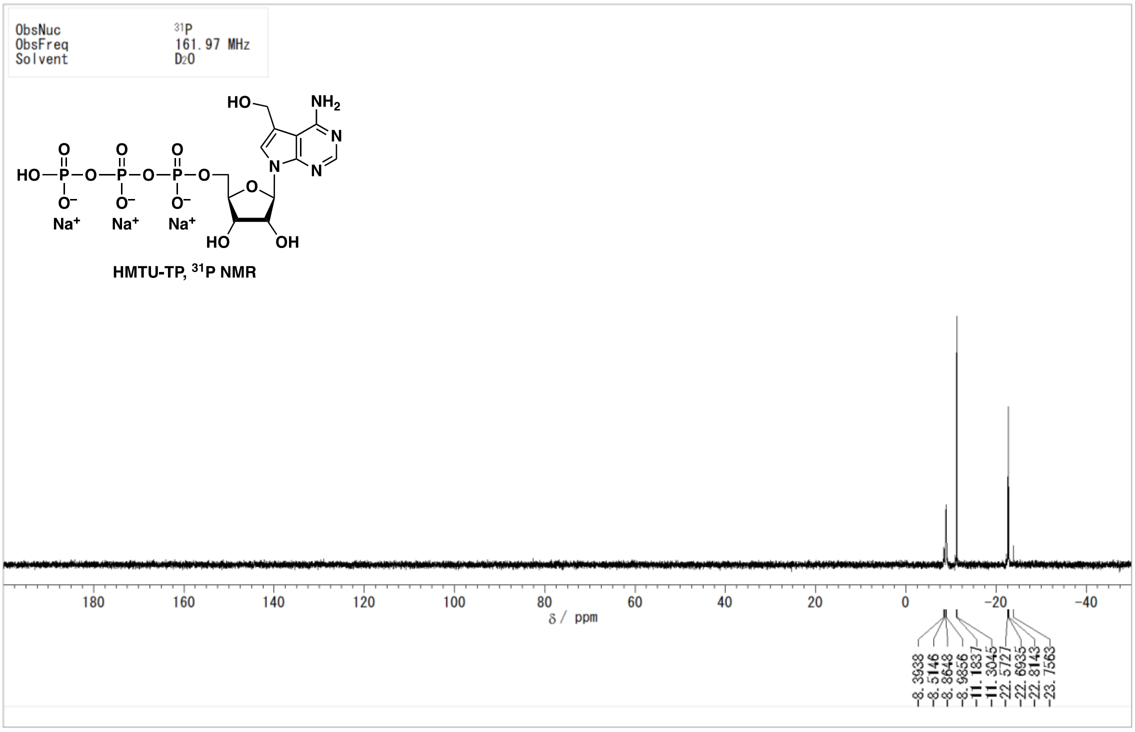
ObsNuc 31P
ObsFreq 161.97 MHz
Solvent D2O
HO
NH2
N
O
HO
P
O
Na+
HMTU-TP, 31P NMR
180
160
140
120
100
80
60
40
20
0
−20
−40
δ / ppm
−8.3938
−8.5146
−8.8648
−8.9856
−11.1837
−11.3045
−22.5727
−22.6935
−22.8143
−23.7563
